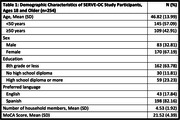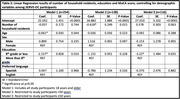# Formal education, household size and cognitive outcomes among Latino individuals: Data from the Skills‐Based Educational strategies for Reduction of Vascular Events in Orange County, Ca (SERVE OC) Trial

**DOI:** 10.1002/alz.088575

**Published:** 2025-01-09

**Authors:** Alissa Kurzman, Xueting Ding, Bruce Albala, Jeffrey Wing, Darnisha Draughter, Aryanna Chavez, Desiree Gutierrez, Bernadette Boden‐Albala

**Affiliations:** ^1^ University of California Irvine, Irvine, CA USA; ^2^ Ohio State University, Columbus, OH USA

## Abstract

**Background:**

In the United States, Latino communities are disproportionately impacted by Alzheimer’s disease and related dementias (ADRDs)^1^ with 11.1% and 5.3% living with mild cognitive impairment and mild dementia due to ADRDs, respectively.^2^ Education is known to be associated with adults’ cognitive function; greater amounts of formal education predict lower risk of dementia in late‐life.^3^ The Montreal Cognitive Assessment (MoCA) is dependent on the educational level of participants, recommending a 1‐point correction for ≤12 years of education when scoring the assessment. However, additional adjustments may be necessary for minority populations with average education levels ≤12 years. Less is known about household size, as a proxy of social support, and cognition. In this study we aim to evaluate education and household size with cognitive outcomes of Hispanic/Latino self‐identifying individuals residing in Southern California.

**Methods:**

Baseline data from the Skills‐Based Educational strategies for Reduction of Vascular Events in Orange County (SERVE OC) Trial were used (Grant Number P50MD017366). Study participants (n = 254) were restricted to individuals ≥18 years who self‐identified as Hispanic/Latino and completed the MoCA 8.3 in English or Spanish at baseline. MoCA scores were adjusted for education, per instrument protocol. Multivariable linear regressions estimated associations between number of household residents, education, and MoCA score, overall and by age (<50 and ≥50 years), controlling for gender and preferred language.

**Results:**

Participants were an average of 46.8 years (SD = 13.99) with 49.2%≥50 years; 67.2% of participants were female and 64.5% had ≤8 years of formal education. Average MoCA scores were 22.20 (SD = 3.71) and 20.67 (SD = 4.62) for individuals <50 and ≥50years respectively. For individuals ≥50 years, having ≤8 years of formal education was significantly associated with lower MoCA scores (B = ‐3.22; p<0.05) and with number of household residents trending towards intact cognition (B = 0.578, p = 0.063). For individuals <50 years, number of household residents is a significant predictor of MoCA score (B = ‐0.618, p<0.05).

**Conclusion:**

Understanding the impact of education and household size is important when assessing memory/cognitive outcomes of Latino individuals. Research is needed to better understand if/how the MoCA should be further adjusted in underrepresented populations and the impact of household composition and dynamics on cognitive impairment.